# The role of competing endogenous RNA network in the development of hepatocellular carcinoma: potential therapeutic targets

**DOI:** 10.3389/fcell.2024.1341999

**Published:** 2024-01-31

**Authors:** Ziwei Tang, Xue Li, Yanfeng Zheng, Jin Liu, Chao Liu, Xia Li

**Affiliations:** ^1^ The Ninth People’s Hospital of Chongqing, Chongqing, China; ^2^ School of Basic Medical Sciences, Chengdu University of Traditional Chinese Medicine, Chengdu, China; ^3^ Chongqing Medical and Pharmaceutical College, Chongqing, China; ^4^ Chongqing Chemical Industry Vocational College, Chongqing, China

**Keywords:** hepatocellular carcinoma, competing endogenous RNA, traditional Chinese medicine, progress, therapy

## Abstract

The current situation of hepatocellular carcinoma (HCC) management is challenging due to its high incidence, mortality, recurrence and metastasis. Recent advances in gene genetic and expression regulation have unveiled the significant role of non-coding RNA (ncRNA) in various cancers. This led to the formulation of the competing endogenous RNA (ceRNA) hypothesis, which posits that both coding RNA and ncRNA, containing miRNA response elements (MRE), can share the same miRNA sequence. This results in a competitive network between ncRNAs, such as lncRNA and mRNA, allowing them to regulate each other. Extensive research has highlighted the crucial role of the ceRNA network in HCC development, impacting various cellular processes including proliferation, metastasis, cell death, angiogenesis, tumor microenvironment, organismal immunity, and chemotherapy resistance. Additionally, the ceRNA network, mediated by lncRNA or circRNA, offers potential in early diagnosis and prevention of HCC. Consequently, ceRNAs are emerging as therapeutic targets for HCC. The complexity of these gene networks aligns with the multi-target approach of traditional Chinese medicine (TCM), presenting a novel perspective for TCM in combating HCC. Research is beginning to show that TCM compounds and prescriptions can affect HCC progression through the ceRNA network, inhibiting proliferation and metastasis, and inducing apoptosis. Currently, the lncRNAs TUG1, NEAT1, and CCAT1, along with their associated ceRNA networks, are among the most promising ncRNAs for HCC research. However, this field is still in its infancy, necessitating advanced technology and extensive basic research to fully understand the ceRNA network mechanisms of TCM in HCC treatment.

## 1 Introduction

Hepatocellular carcinoma (HCC) is among of the most prevalent malignant tumors, characterized by diverse etiologies, significant morbidity and mortality, and a poor survival prognosis. Over half of the global HCC cases are reported in Asia (Singal et al., 2020). The insidious nature of HCC often results in the absence of early-stage clinical manifestations, leading to its frequent oversight. Consequently, most HCC cases are diagnosed at an advanced stage, beyond the optimal timeframe for surgical intervention, and with limited treatment options. Despite recent advancements in early diagnosis and treatment, the clinical outcomes for HCC remain poor, largely due to high rates of recurrence and metastasis (Ghouri et al., 2017).

Addressing this challenge, it is critical to develop effective strategies for the early screening and monitoring of HCC. While Alpha-fetoprotein (AFP) is a widely used diagnostic biomarker for HCC, its limited sensitivity and specificity, as evidenced by the presence of AFP-negative HCC patients, indicates a need for improvement (Wang and Wei, 2020). The advancement of systems biology and bioinformatics technologies has led to the development of new biomarkers, such as alpha-fetoprotein lectin 3 (AFP-L3), des-gamma-carboxyprothrombin (DCP), and glypican-3 (GPC3). However, the clinical applicability of these biomarkers requires further evaluation (Pinero et al., 2020). Therefore, exploring novel potential biomarkers is essential to enhance diagnostic methods and improve treatment outcomes for HCC.

Non-coding RNA (ncRNA), including microRNA (miRNA), long non-coding RNA (lncRNA), and circular RNA (circRNA), constitute a major portion of the human transcriptome (Chan and Tay, 2018), with only 1%–2% being protein-coding mRNAs (Carninci et al., 2005; Djebali et al., 2012). This discovery has sparked significant interest in studying the functions of ncRNAs. These RNAs have been identified as key players in various biological processes, particularly in cancer development. MiRNA, a small ncRNA of approximately 22 nucleotides, post-transcriptionally regulates gene expression by binding to miRNA response elements (MRE), leading to gene degradation or translation inhibition (Bartel, 2004; 2018). Aberrant miRNA expression is closely linked to tumorigenesis (Di Leva et al., 2014), exhibiting both carcinogenic and tumor-suppressive effects in a dosage-dependent manner (Svoronos et al., 2016). Initial research on miRNA focused on their unidirectional regulation of target genes. However, further investigation has revealed intricate co-regulatory networks between non-coding and coding RNAs, leading to the competing endogenous RNA (ceRNA) hypothesis (Salmena et al., 2011). The ceRNA network, implicated in tumor cell proliferation, metastasis, apoptosis, angiogenesis, chemotherapy resistance, stem cell regulation, tumor immunity (Chen et al., 2022c). Comprehensive research on ceRNA function in HCC could identify potential biomarkers for early diagnosis and novel therapeutic targets. CeRNAs, encompassing both ncRNA and mRNA, present multifaceted targets. While Western medicine often faces challenges in achieving multi-target effects, traditional Chinese medicine (TCM) has shown significant advantages due to its complex composition and diverse targets. Thus, this study rigorously reviews the role of ceRNAs in HCC development and their potential as therapeutic targets, offering a novel perspective for researching TCM and non-TCM compound interventions in HCC (Supplementary Figure).

## 2 Overview of ceRNA regulation

**FIGURE 1:**
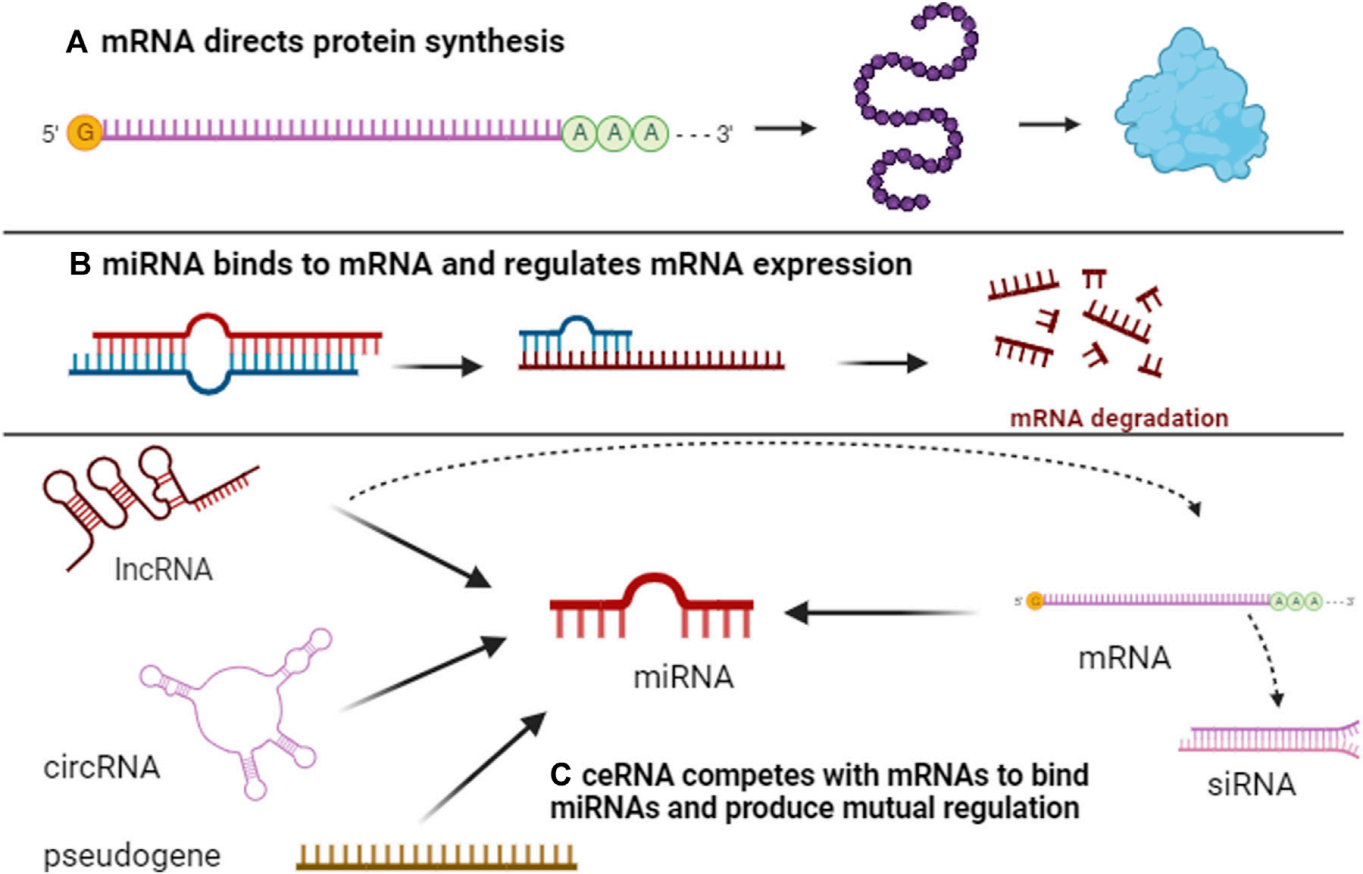
Overview of ceRNA networks. Initially, protein-coding gene mRNAs serve to carry genetic information, encode for protein synthesis, and execute biological functions **(A)**. Subsequently, mRNAs interact with miRNAs through MRE, resulting in either mRNA degradation or inhibition of protein synthesis **(B)**. Concurrently, ncRNAs, including lncRNAs, circRNAs, and pseudogenes, also possess MREs and establish a competitive dynamic with mRNAs. When ncRNAs bind more frequently to miRNAs, the latter’s binding to mRNAs is reduced, consequently mitigating mRNA degradation by miRNAs and facilitating gene interaction **(C)**.

To our knowledge, protein-coding genes are predominantly responsible for genome functionality. Research indicates that the number of protein-coding genes in *C. elegans* (*Caenorhabditis elegans*) is analogous to that in humans (Baltimore, 2001). However, *C. elegans* remains a simpler organism, suggesting that protein-coding genes alone do not dictate the complexity of higher organisms. Additionally, the human genome contains approximately 30 times more genes than *C. elegans*, underscoring the crucial role of non-coding genome components in the sophistication and diversity of higher eukaryotes compared to simpler organisms (Costa, 2008; Mattick, 2009). This realization has intensified interest in non-coding genes within human genomics. Recent studies have elucidated notable changes in non-coding genes through comprehensive analyses of cancer genomes and transcriptomes (Futreal et al., 2004; Stratton et al., 2009; Beroukhim et al., 2010), fueling a surge in non-coding gene research in oncology. Despite evidence of a strong association between lncRNAs and certain cancer-related pathological processes, the broader implications of non-coding genes' functions and the potential non-coding roles of coding genes remain largely unexplored (Nagano and Fraser, 2011). Consequently, researchers are increasingly investigating the interactions between coding and non-coding genes. Emerging studies suggest that RNA molecules can modulate each other’s expression by competing for a limited supply of miRNA under specific conditions (Seitz, 2009; Poliseno et al., 2010). This led to the formulation of the ceRNA hypothesis, postulating that various RNA transcripts interact through a unique mechanism mediated by MRE, influencing the physiological or pathological progression of organisms (Figure 1).

## 3 Characteristics and biological functions of ceRNA

In ceRNA regulation, miRNAs are the focal point of competition, whereas coding genes' mRNAs or non-coding genes' lncRNA or circRNA with MRE are collectively identified as ceRNAs (Shi et al., 2021b; Chen et al., 2022c). These ceRNAs exhibit distinct biological characteristics and functions. While protein-coding genes' mRNAs are typically the downstream target genes in ceRNA networks and are primary targets for non-coding RNA interventions, they can also influence other mRNAs through MRE, creating a complex regulatory network and diversifying functions in biological and pathological contexts (Tay et al., 2014). MiRNA, approximately 22 nucleotides in length, regulate target genes at the transcriptional level by binding to MRE on target genes, leading to their downregulation or silencing (Bartel, 2009). MiRNA are known to modulate over 50% of mRNAs (Friedman et al., 2009; Thomas et al., 2010), playing a pivotal role in growth and the progression of various diseases, particularly cancer (Lu et al., 2008). LncRNAs, acting as sponges, guides, and regulators, influence transcription, chromatin structure, and histone modification, and are crucial in numerous biological processes, including epigenetic regulation, cell differentiation, motility, and apoptosis, thus drawing significant attention in gene regulation research (Abbasifard et al., 2020). CircRNAs, notable for their stable, ring-closed structure (Dong et al., 2023), are rich in MRE and excel as molecular "sponges", effectively countering miRNA-mediated inhibition (Kristensen et al., 2019). Pseudogenes, often disrupted by premature termination codons, frame-shifting mutations, insertions, or deletions, are traditionally regarded as "non-functional", "redundant", or "evolutionary remnants" (D'Errico et al., 2004). However, due to their high sequence similarity to miRNA-regulated known genes, pseudogenes actively compete in and contribute to ceRNA network regulation (Birney et al., 2007).

## 4 The role of ceRNA networks in the development of HCC

**FIGURE 2:**
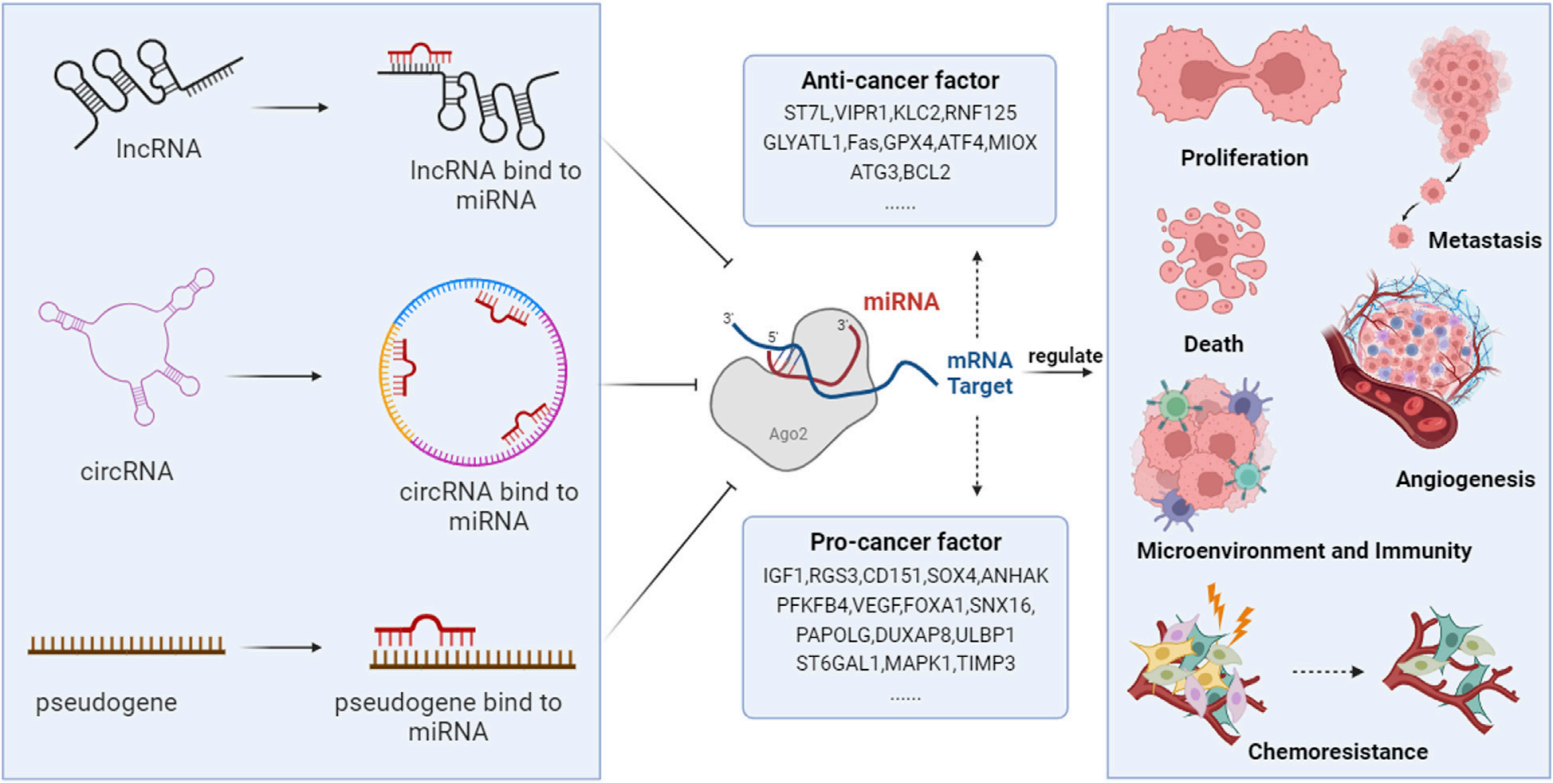
The ceRNA networks are involved in the development of HCC. NcRNAs, including lncRNAs, circRNAs, and pseudogenes, compete with coding RNAs for miRNA binding, as they all possess MRE. Through this mechanism, they regulate the degradation of miRNAs on target genes, thus modulating the expression of genes that are vital in controlling HCC’s proliferation, metastasis, angiogenesis, cell death, host immunity, tumor microenvironment, and chemotherapy resistance.

Advancements in epigenetic technologies have demonstrated that ncRNAs and ceRNA networks play a critical role in the pathogenesis of HCC. They are involved in regulating processes such as proliferation, invasion, metastasis, cell death, angiogenesis, the immune-tumor microenvironment, and chemoresistance (Figure 2).

### 4.1 Regulation of HCC cell proliferation

Uncontrolled proliferation is a primary characteristic of tumor cells, and numerous studied have indicated that ceRNA networks are crucial in regulating this proliferation. Non-coding RNAs regulate by competitively binding miRNAs to these proliferation-related genes, thereby affecting their transcription and expression. IGF1, a key cell growth-related factor, regulates growth, development, and the cell growth cycle, promoting cell proliferation, division, and regulating metabolism. Its expression was notably increased in various tumors (De Santi et al., 2016). LncRNA TUG1 acted as a ceRNA, sponging miR-1-3p to enhance IGF1 expression, thus facilitating HCC cell proliferation (Tang et al., 2022a). Transcription factors such as FOXA1 and E2F1 are known for their roles in carcinogenesis by modulating gene transcription and participating in processes like cell proliferation and differentiation (He et al., 2017). Studies showed that lncRNA MCM3AP-AS1 interacted with miR-194–5p, resulting in upregulated FOXA1, a target of miR-194–5p, which reduced HCC cell proliferation and cell cycle progression (Wang et al., 2019). Similarly, lncRNA APUE countered the inhibitory effect of miR-20b on E2F1, leading to increased E2F1 levels that foster cell cycle progression and tumor growth (Li et al., 2021c). Additionally, some ncRNAs induced protein signaling *via* the ceRNA network to boost HCC cell proliferation, such as UBE2MP1/miR-145–5p/RGS3 (Hao et al., 2022), lncRNA *miat*/miR-22–3p/Sirt1 (Zhao et al., 2019), and LINC00152/miR-143/KLC2 (Pellegrino et al., 2022). Furthermore, the pseudogene DUXAP8 encouraged HCC cell differentiation by modifying kinase function through DUXAP8/miR-490–5p/BUB1 (Zhang et al., 2020a). However, when certain lncRNAs target miRNAs of oncogenes, they often inhibit HCC proliferation, as observed with lncRNA MIR31HG (Yan et al., 2018) and lncRNA-AC079061.1 (Lin et al., 2022).

### 4.2 Regulation of HCC cell invasion and metastasis

The aggressive and migratory behavior of tumors contributes to drug resistance and increased mortality rates. Tumor metastasis involves cell adhesion, extracellular matrix degradation, and phenotypic changes in tumor cells. NcRNAs can regulate HCC invasion by acting on relevant genes or signaling pathways through the ceRNA network. The epithelial-mesenchymal transition (EMT) is closely linked to HCC recurrence and metastasis, receiving increasing attention (Tam and Weinberg, 2013; Xie and Diehl, 2013). Signaling factors like CD151, SOX4, and FOSL2 are critical in EMT acquisition in HCC cells, enabling some ncRNAs to bind target miRNAs through sponging, thereby promoting HCC invasion and metastasis. Studies have demonstrated that PI3KC2 and LAMC1, as mRNAs, sequester miR-124, enhancing CD151 expression and facilitating HCC invasion and metastasis (Liu et al., 2016a). Circ-TLK1 and LncRNA HOSD-AS1 increased SOX4 expression by sponging miR-138–5p and miR-130a-3p, respectively, affecting EMT regulation and promoting aggressive HCC cell behavior (Wang et al., 2017; Lu Y. et al., 2022b). Circ0003998 bound miR-143–3p as a ceRNA both *in vitro* and *in vivo,* mitigating the inhibitory effect on EMT stimulator FOSL2 and advancing HCC invasion and metastasis (Song et al., 2020). Additionally, ANHAK influenced the EMT process through TGF-β, and circ0008194 acted as a sponge in the ceRNA network to promote HCC invasion (Liu et al., 2022c). RTKN2 and the Wnt/β-catenin signaling pathway contributed to tumor malignancy (Zhang et al., 2023). Has-circRNA-104348 indirectly regulated RTKN2 by binding miR-187–3p, promoting HCC lung metastasis *in vivo* (Huang et al., 2020). CeRNA networks are also involved in pathways like HCC cell metabolism and proliferation of metastatic foci, furthering HCC invasion and metastasis (Wei et al., 2020; Lai et al., 2021). Conversely, some ncRNA-mediated ceRNA networks inhibited HCC invasion by activating JAK1 polyubiquitination, increasing growth transcriptional repressors' expression, and enhancing glutamine metabolism (Lu et al., 2020; Bao et al., 2022; Peng et al., 2022b).

### 4.3 Regulation of HCC cell death

Cell death can be classified into programmed and non-programmed (necrosis) categories. Programmed cell death is an active process that can be inhibited by cell signaling inhibitors and includes apoptosis, autophagy, ferroptosis, mitotic catastrophe, bloat death, senescence-like cell death, and parapoptosis (D'Arcy, 2019; Kopeina and Zhivotovsky, 2022). Apoptosis, a well-researched programmed death mode, involves three signaling pathways, the death receptor, mitochondrial, and endoplasmic reticulum stress pathways, all culminating in caspase activation leading to apoptosis (Green, 2019). Autophagy, characterized by numerous autophagic vesicles in the cytoplasm, involves the fusion of these vesicles with lysosomal membranes for enzymatic degradation within lysosome (Kocaturk et al., 2019). Excessive autophagy results in autophagic cell death (Liu et al., 2023). Ferroptosis, a novel iron-dependent programmed cell death mode, primarily induces cell death through lipid peroxidation, catalyzed by divalent iron or ester oxygenase and affecting unsaturated fatty acids on cell membranes (Dixon et al., 2012). Conversely, necrosis, an uncontrolled cell death process unaffected by cell signaling inhibitors, occurs under physical or chemical environmental stimuli. Necrosis is marked by cell membrane disruption, cellular and organelle edema, absence of chromatin agglutination, and is often associated with inflammation and surrounding tissue damage (Park et al., 2023).

The ceRNA network in the apoptotic process of HCC cells exhibits varied roles depending on its target mRNAs, often leading to pro-apoptotic effects if targeting factors such as Fas (Zhang et al., 2017). When targeting apoptotic regulators, it inhibited apoptosis activation, as seen in the lncRNA SNHG6-003/miR-26a/b/TAK1 network (Cao et al., 2017). Similarly, acting on the FAK/Src/ERK pathway, it could inhibit apoptosis in HCC cells, exemplified by the lncRNA DBH-AS1/miR-138/FAK network (Bao et al., 2018).

Ferroptosis, characterized by iron-dependent lipid peroxidation, stands out as a distinct cell death process, differing from other regulated cell death forms in morphology, genetics, and biochemistry (Stockwell et al., 2017). The ceRNA network plays a significant role in the ferroptosis process in HCC cells. Recent studies found that ketamine induced ferroptosis in HCC cells through the Lnc-PVT1/miR-214–3p/GPX4 axis, reducing cell viability and proliferation (He et al., 2021). Knockdown of lncRNA HULC impacted HCC cell death, primarily by inducing ferroptosis *via* miR-3200-5p-mediated ATF4 targeting (Guan et al., 2022). LncRNA NEAT1 competitively bound to miR-362–3p, diminishing MIOX repression by miR-362–3p and increasing HCC cell susceptibility to ferroptosis (Zhang et al., 2022c). It has been suggested that lncRNA DNAJC27-AS1 and PPIF competitively bound miR-23b-3p, leading to an interaction that downregulates ferroptosis in HCC cells (Yang et al., 2023).

Autophagy involves engulfing cytoplasmic proteins or organelles and fusing them with lysosomes to form autophagic lysosomes, which degrade the contents, facilitating metabolism and cell renewal (Lee and Jang, 2015). Research indicated that autophagy was triggered in HCC under various stress conditions, such as hypoxia, nutrient deprivation, and chemotherapy, contributing to tumorigenesis (Codogno and Meijer, 2013; Cui et al., 2013). Autophagy-associated proteins ATG3 and ATG5 were involved in the lncRNA PVT1 and lncRNA HNF1A-AS1-mediated ceRNA network that promoted autophagy in HCC cells (Liu et al., 2016b; Yang et al., 2019).

### 4.4 Regulation of HCC angiogenesis

Angiogenesis is essential for the growth and progression of HCC (Morse et al., 2019). This process occurs from the initial tumor stage through to metastasis. Vascular endothelial growth factor (VEGF) is a key angiogenic factor and is elevated in most malignant tumors (Han et al., 2021). The ceRNA network facilitates angiogenesis in HCC cells by targeting miRNAs that enhance VEGF and its receptor expression. For instance, lncRNA NEAT1 regulated angiogenesis in HCC by competing with VEGF for miR-125a-5p binding and modulating the AKT/mTOR and ERK pathways (Guo et al., 2022). Similarly, lncRNA MYLK-AS1 advanced HCC angiogenesis through the miR-424–5p/E2F7/VEGFR-2 pathway (Teng et al., 2020). In addition, ceRNA networks contributed to HCC angiogenesis by stimulating pro-angiogenic factors, such as the lncRNA MALAT1/miR-3064–5p/FOXA1 network (Zhang et al., 2020b). Moreover, research showed that cells lacking LINC00488 or with overexpressed miR-330–5p could suppress *in vivo* tumor growth and angiogenesis, highlighting LINC00488’s ceRNA role in HCC (Gao et al., 2019).

### 4.5 Regulation of HCC microenvironment and host immunity

The tumor microenvironment (TME) is a dynamic internal environment that fosters tumor progression. Research indicates that altering the TME is a potential mechanism of action for anticancer drugs (Hanahan and Coussens, 2012). NcRNAs are pivotal in regulating innate and adaptive immunity (Carpenter et al., 2013). In HCC, the ceRNA network modulates the function of various immune cells, impacting host immunity and the TME (Zuo et al., 2021). For example, the lncRNA MIR4435-2HG/hsa-miR-1-3p/MMP9/hsa-miR-29–3p/DUXAP8 association with immune cell infiltration correlated with higher risk and worse outcomes in HCC (Zhang et al., 2021). Notably, NK cell infiltration is linked to tumor immune evasion. The LINC00638/miR-4732–3p/ULBP1 and lncRNA PART1/hsa-miR-125a-5p/CDK5R2 networks influenced NK cell function, fostering immune escape and vascular invasion in HCC (Qi et al., 2021; Cai et al., 2022). Additionally, the lncRNA MIR155HG enhanced tumor immune evasion induced by lipopolysaccharide through the miR-223/STAT1 axis, acting as a ceRNA (Peng et al., 2022a). Beyond NK cells, the ceRNA network also affects functional changes in B cells, T cells, and macrophages. For instance, LINC00261, by sponging miR105–5p, upregulated SELL expression in HCC, leading to altered B cell, T cell, and macrophage function (Song et al., 2022). Analysis of immune infiltration revealed that the lncRNA GSEC/miR-101–3p/SNX16/PAPOLG axis might influence macrophage polarization, a key factor in poor HCC prognosis (Hu et al., 2022). The ceRNA network related to CFHR4 regulated its expression, impacting the tumor immune microenvironment. High CFHR4 expression was significantly associated with increased dendritic cells, mast cells, neutrophils, NK cells, and T cells, underscoring its role in immune cell infiltration in HCC (Yu et al., 2022).

### 4.6 Regulation of chemoresistance in HCC

Chemotherapy is a pivotal treatment for tumors, yet its efficacy and side effects are influenced by patient-specific factors and tumor types. In HCC, characterized by high proliferation, metastasis, and drug resistance, some patients experience rapid deterioration post-chemotherapy. The role of ceRNA in cancer treatment resistance has become increasingly recognized.

Oxaliplatin, a primary chemotherapy agent for HCC, faces challenges with ceRNA-related resistance. Research indicated that lncRNA TINCR acted as a miR-195–3p sponge, reducing its inhibition of ST6GAL1. This triggers the NF-κB signaling pathway, thereby enhancing oxaliplatin resistance (Mei et al., 2022). Overexpression of lncRNA NR2F1-AS1 in oxaliplatin-resistant HCC tissues has been observed. It bound to miR-363, increasing ABCC1 expression and contributing to oxaliplatin resistance (Huang et al., 2018). Additionally, lncRNA KCNQ1OT1 regulated ABCC1 expression *via* miR-7-5p, promoting resistance in HepG2 and Huh7 cells (Hu et al., 2018). Thus, ABCC1 emerges as a crucial ceRNA target in combating oxaliplatin resistance in HCC. Moreover, lncRNA PCGEM1, HULC, and circFBXO11 have been implicated in oxaliplatin resistance through their respective ceRNA networks (Li et al., 2020; Chen J. et al., 2021a; Li et al., 2021b).

Cisplatin, another widely-used chemotherapy drug, has been the focus of studies on ceRNA network-related resistance in HCC. The lncRNA LEF1-AS1/miR-10a-5p/MSI1 network has been identified as a key player in cisplatin resistance. Through both *in vitro* and *in vivo* methods like gene knockdown or overexpression, it was found that LEF1-AS1 enhanced cisplatin resistance in HCC cells by targeting miR-10a-5p to elevate MSI1 expression and actuate the AKT signaling pathway (Gao et al., 2021).

Sorafenib is a leading drug for treating advanced HCC, extending the median survival of HCC patients by 12.3 months (Kudo et al., 2018). Yet, its effectiveness wanes due to resistance development. The upregulation of the pseudogene-derived lncRNA DUXAP8 has been linked to HCC progression and reduced sorafenib efficacy, principally through DUXAP8’s modulation of MAPK1 *via* miR-584–5p, thus activating the MAPK/ERK pathway (Liu et al., 2021). Additionally, circRNA-001241, by sequestering miR-21–5p, enhanced TIMP3 expression, contributing to sorafenib resistance in HCC (Yang and Wu, 2021). Furthermore, the lncRNAs TRERNA1, HANR, and TTN-AS1 were involved in sorafenib resistance, primarily *via* the RAS/Raf/MEK/ERK axis, promoting autophagy, and activating the PTEN/Akt pathway (Shi et al., 2020; Zhou et al., 2021c; Song et al., 2021). Conversely, circITCH and lncRNA FOXD2-AS1 may inhibit sorafenib resistance by targeting miR-20b-5p and miR-150–5p, underscoring a potential positive role of the ceRNA network in combating HCC chemotherapy resistance (Sui et al., 2019; Li et al., 2023). Further details about the ceRNA network in HCC are outlined in Table 1.

**TABLE 1:** Impact of ceRNA network on HCC.

Category	CeRNA network	Function on HCC	References
Proliferation	LINC00152/miR-143/KLC2	Promote proliferation	Pellegrino et al. (2022)
	lncRNA TUG1/miR-1-3p/IGF1	Promote proliferation	Tang et al. (2022a)
	lncRNA MCM3AP-AS1/miR-194–5p/FOXA1	Promote proliferation	Wang et al. (2019)
	lncRNA *miat*/miR-22–3p/Sirt1	Promote proliferation	Zhao et al. (2019)
	lncRNA APUE/miR-20b/E2F1	Promote proliferation	Li et al. (2021c)
	UBE2MP1/miR-145–5p/RGS3 G	Promote proliferation	Hao et al. (2022)
	DUXAP8/miR-490–5p/BUB1	Promote proliferation	Zhang et al. (2020a)
	lncRNA MIR31HG/miR-575/ST7L	Inhibit proliferation	Yan et al. (2018)
	lncRNA-AC079061.1/miR-765/VIPR1	Inhibit proliferation	Lin et al. (2022)
Invasion and Metastasis	PI3KC2/miR-124/CD151	Promote invasion and metastasis	Liu et al. (2016a)
	circ-TLK1/miR-138–5p/SOX4	Promote invasion and metastasis	Lu et al. (2022b)
	circ0008194/miR-190a/ANHAK	Promote invasion and metastasis	Liu et al. (2022c)
	circ104348/miR-187–3p/RTKN2	Promote invasion and metastasis	Huang et al. (2020)
	circ0003998/miR-143–3p/FOSL2	Promote invasion and metastasis	Song et al. (2020)
	LINC01572/miR-195–5p/PFKFB4	Promote invasion and metastasis	Lai et al. (2021)
	lncRNA HOXD-AS1/miR-130a-3p/SOX4	Promote invasion and metastasis	Wang et al. (2017)
	lncRNA MFI2-AS1/miR-134/FOXM1	Promote invasion and metastasis	Wei et al. (2020)
	circPTTG1IP/miR-16–5p/RNF125	Inhibit invasion and metastasis	Peng et al. (2022b)
	LINC00675/miR-942–5p/GFI1	Inhibit invasion and metastasis	Lu et al. (2020)
	PLGLA/miR-324–3p/GLYATL1	Inhibit invasion and metastasis	Bao et al. (2022)
Cell death	STARD13/miR-340, miR-448, miR-374, miR-203, let-7, miR-216b, miR-316, miR-23, miR-153/Fas	Promote apoptosis	Zhang et al. (2017)
	lncRNA SNHG6-003/miR-26a/b/TAK1	Inhibit apoptosis	Cao et al. (2017)
	lncRNA DBH-AS1/miR-138/FAK/Src/ERK pathway	Inhibit apoptosis	Bao et al. (2018)
	Lnc-PVT1/miR-214–3p/GPX4	Promote ferroptosis	He et al. (2021)
	lncRNA NEAT1/miR-362–3p/MIOX	Promote ferroptosis	Zhang et al. (2022c)
	lncRNA HULC/miR-3200–5p/ATF4	Inhibit ferroptosis	Guan et al. (2022)
	lncRNA DNAJC27-AS1/miR-23b-3p/PPIF	Inhibit ferroptosis	Yang et al. (2023)
	lncRNA PVT1/miR-365/ATG3	Promote autophagy	Yang et al. (2019)
	lncRNA HNF1A-AS1/miR-30b/ATG5	Promote autophagy	Liu et al. (2016b)
Angiogenesis	lncRNA NEAT1/miR-125a-5p/VEGF	Promote angiogenesis	Guo et al. (2022)
	lncRNA MYLK-AS1/miR-424–5p/E2F7	Promote angiogenesis	Teng et al. (2020)
	lncRNA MALAT1/miR-3064–5p/FOXA1	Promote angiogenesis	Zhang et al. (2020b)
	LINC00488/miR-330–5p/TLN1	Promote angiogenesis	Gao et al. (2019)
Microenvironment and host immunity	LINC00261/miR105–5p/SELL	B cell dysfunction	Song et al. (2022)
	lncRNA GSEC/miR-101–3p/SNX16/PAPOLG	Macrophage polarization	Hu et al. (2022)
	lncRNA MIR4435-2HG/miR-1-3p/MMP9; lncRNA MIR4435-2HG/miR-29–3p/DUXAP8	Immune cell infiltration	Zhang et al. (2021)
	LINC00638/miR-4732–3p/ULBP1	NK cell infiltration, immune escape	Qi et al. (2021)
	lncRNA MIR155HG/miR-223/STAT1	Tumor immune escape	Peng et al. (2022a)
	lncRNA PART1/miR-125a-5p/CDK5R2	NK cells infiltration	Cai et al. (2022)
Chemoresistance	lncRNA TINCR/miR-195–3p/ST6GAL1	Promote oxaliplatin resistance	Mei et al. (2022)
	lncRNA NR2F1-AS1/miR-363/ABCC1	Promote oxaliplatin resistance	Huang et al. (2018)
	lncRNA KCNQ1OT1/miR-7-5p/ABCC1	Promote oxaliplatin resistance	Hu et al. (2018)
	lncRNA PCGEM1/miR-129–5p/ETV1	Promote oxaliplatin resistance	Chen et al. (2021a)
	lncRNA HULC/miR-383–5p/VAMP2	Promote oxaliplatin resistance	Li et al. (2021b)
	circFBXO11/miR-605/FOXO3	Promote oxaliplatin resistance	Li et al. (2020)
	lncRNA LEF1-AS1/miR-10a-5p/MSI1	Promote cisplatin resistance	Gao et al. (2021)
	lncRNA DUXAP8/miR-584–5p/MAPK1	Promote sorafenib resistance	Liu et al. (2021)
	circRNA-001241/miR-21–5p/TIMP3	Promote sorafenib resistance	Yang and Wu (2021)
	lncRNA HANR/miR-29b/ATG9A	Promote sorafenib resistance	Shi et al. (2020)
	lncRNA TRERNA1/miR-22–3p/NRAS	Promote sorafenib resistance	Song et al. (2021)
	lncRNA TTN-AS1/miR-16–5p/CyclinE1	Promote sorafenib resistance	Zhou et al. (2021c)
	circITCH/miR-20b-5p/PTEN	Inhibit sorafenib resistance	Li et al. (2023)
	lncRNA FOXD2-AS1/miR-150–5p/TMEM9	Inhibit sorafenib resistance	Sui et al. (2019)

## 5 The role of ceRNA in the clinical diagnosis and prognosis of HCC

AFP is the most widely used serum biomarker for HCC diagnosis, crucial for early detection and disease monitoring. However, its diagnostic sensitivity is limited, specificity is suboptimal, and approximately 30% of early-stage HCC patients do not exhibit elevated AFP levels (Luo et al., 2020). Although advancements in liver ultrasound, computed tomography (CT), and magnetic resonance imaging (MRI) have been significant, they are not suitable for routine screening in high-risk HCC individuals. Therefore, the identification of circulating biomarkers with high sensitivity, specificity, and ease of longitudinal monitoring is imperative.

With advancements in the study of the ceRNA network, its importance in the clinical diagnosis and prognosis of HCC has become evident. Abnormal expression of several ncRNAs and their associated ceRNA networks is often observed in HCC tissues and circulating fluids. One study utilized bioinformatics to construct a robust ceRNA network from public datasets, demonstrating the ZFAS1/hsa-miR-150–5p/GINS1 network’s significant contribution to HCC diagnosis and prognosis (Chen et al., 2022c). Additionally, research identified lncRNA MYCNOS, DLX6-AS1, LINC00221, CRNDE, and mRNAs CCNB1 and SHCBP1 as closely linked to HCC patient prognosis (Long et al., 2019). These findings underscore the potential of the ceRNA network in enhancing early diagnosis and predicting the outcome of HCC (Table 2).

**TABLE 2:** Potential clinical application of ceRNA networks in HCC.

CeRNA network	Location	Clinical value	References
lncRNA TUG1/miR-524–5p/SIX1	Tissue	Diagnostic and prognostic marker	Lin et al. (2020), Lu et al. (2022a)
lncRNA NEAT1/miR-9-BGH3	Tissue and serum	Diagnostic marker	Mohyeldeen et al. (2020), Tripathi et al. (2022)
lncRNA CDKN2BAS/miR-153–5p/ARHGAP18	Tissue	Prognostic marker	Chen et al. (2018)
lncRNA RP11-386G11.10/miR-345–3p/HNRNPU	Tissue	Prognostic marker	Xu et al. (2022)
lncRNA LUCAT1/miR-495- 3p/DLC1	Tissue	Prognostic marker	Wu et al. (2020)
lncRNA ZFPM2-AS1/miR-515–5p/DAPK2	Tissue	Prognostic marker	Wu et al. (2020)
circ0091579/miR-136–5p/TRIM27	Tissue	Diagnostic and prognostic marker	Zhang et al. (2018), Mao et al. (2022)
circ0016788/miR-486/CDK4	Tissue	Diagnostic and prognostic marker	Guan et al. (2019)
circ0016788/miR-506–3p/PAPR14	Tissue	Diagnostic and prognostic marker	Chen et al. (2021c)
circGPR137B/miR-4739/FTO	Tissue	Prognostic marker	Liu et al. (2022b)
circ0004001, circ0004123, circ0075792/targeted miRNA/VEGF/VEGFR, PI3K/AKt, mTOR, Wnt signaling pathways	Serum	Diagnostic marker	Sun et al. (2020)
circ0000437/miR-626/CDKN1B	Tissue and serum	Diagnostic and prognostic marker	Chen et al. (2022a), Li and Liu (2022)
circ0071662/miR-146b-3p/HPGD/NF2	Tissue and serum	Diagnostic and prognostic marker	Abulizi et al. (2019), Wang et al. (2023)

### 5.1 The role of lncRNA-associated ceRNA network in the diagnosis and prognosis of HCC

Numerous studies have highlighted the significance of lncRNA in diagnosing and prognosticating HCC. The lncRNAs TUG1 and NEAT1, recently extensively researched, are emerging as novel biomarkers. TUG1, more effective than AFP for early HCC diagnosis, regulated AFP and served as a prognostic indicator for non-viral HCC (Lin et al., 2020). NEAT1, found to be diminished in hepatitis C and associated HCC, enhanced the diagnostic accuracy of HCC in hepatitis C patients when combined with TUG1, p53, and AFP (Mohyeldeen et al., 2020). TUG1 promoted SIX1 expression, increasing glycolysis and metastasis in HCC cells by interacting with miR-524–5p (Lu et al., 2022a) and activated the JAK/STAT3 pathway by targeting miR-144 (Lv et al., 2018). Additionally, NEAT1 influenced HCC progression by modulating the miR-9-BGH3 axis (Tripathi et al., 2022). Furthermore, the lncRNA CDKN2BAS/miR-153–5p/ARHGAP18 and RP11-386G11.10/miR-345–3p/HNRNPU networks, upregulated in HCC tissues, correlated with larger tumor size, advanced TNM stage, and poorer prognosis in patients (Chen et al., 2018; Xu et al., 2022). Also, a study identified four autophagy-related lncRNAs (LUCAT1, AC099850.3, ZFPM2-AS1, and AC009005.1) as prognostic markers in HCC. A predictive model developed using these lncRNAs was an independent prognostic factor and outperformed other clinicopathologic factors in predicting 1-, 3-, and 5-year survival rates (AUC 0.764, 0.738, and 0.717, respectively). LUCAT1 and ZFPM2-AS1 regulated autophagy in HCC cells through the miR-495–3p/DLC1 and miR-515–5p/DAPK2 axes, respectively (Wu et al., 2020). In summary, the ceRNA networks involving TUG1/miR-524–5p/SIX1, NEAT1/miR-9-BGH3, CDKN2BAS/miR-153–5p/MEK-ERK1/2, RP11-386G11.10/miR-345–3p/HNRNPU, and LUCAT1/miR-495–3p/DLC1 are promising for HCC diagnosis and prognosis.

### 5.2 The role of circRNA-associated ceRNA network in the diagnosis and prognosis of HCC

CircRNAs also hold significant diagnostic value in HCC due to their stable expression, yielding numerous potential biomarkers for early HCC detection. Elevated levels of circ-0091579 in HCC tissues were strongly associated with reduced survival in patients (Zhang et al., 2018). Additionally, circ0091579 acted as an oncogenic factor in HCC development and progression by regulating ceRNA networks, including miR-136–5p/TRIM27 (Mao et al., 2022), miR-1287/PDK2 (Shu et al., 2021), miR-1225–5p/PLCB1 (Zhang et al., 2022a). The ceRNA networks of circ0016788, specifically circ0016788/miR-486/CDK4 and circ0016788/miR-506–3p/PAPR14, are known to significantly enhance HCC proliferation and invasion, presenting notable diagnostic potential for clinical research (Guan et al., 2019; Chen et al., 2021c). Similarly, the downregulated circGPR137B/miR-4739/FTO network indicated a poor prognosis for HCC patients (Liu et al., 2022b). Abnormal circRNA expression in the peripheral blood of HCC patients makes serum samples more conducive for early detection and diagnosis. A clinical study identified 12 distinct circRNAs in HCC patient blood, with three circRNAs (hsa_circ_0004001, hsa_circ_0004123, hsa_circ_0075792) demonstrating high sensitivity and specificity for HCC detection as evidenced by ROC analysis. These circRNAs regulated HCC progression by binding miRNAs and influencing VEGF/VEGFR, PI3K/AKt, mTOR, and Wnt signaling pathways (Sun et al., 2020). Furthermore, circ0000437 and circ0071662, aberrantly expressed in both HCC tissues and serum, are linked to tumor-node-metastasis (TNM) grade, differentiation degree, tumor size, and BCLC stage through their ceRNA networks circ0000437/miR-626/CDKN1B (Li and Liu, 2022) and circ0071662/miR-146b-3p/HPGD/NF2 (Abulizi et al., 2019) underlining their substantial clinical diagnostic relevance (Chen et al., 2022a; Wang et al., 2023). Therefore, the ceRNA network not only contributes to the pathogenesis of HCC but also plays a pivotal role in the clinical diagnosis and prognosis of HCC patients, offering biomarkers for early detection and ongoing monitoring.

## 6 ceRNAs as the therapeutic targets in HCC

**FIGURE 3:**
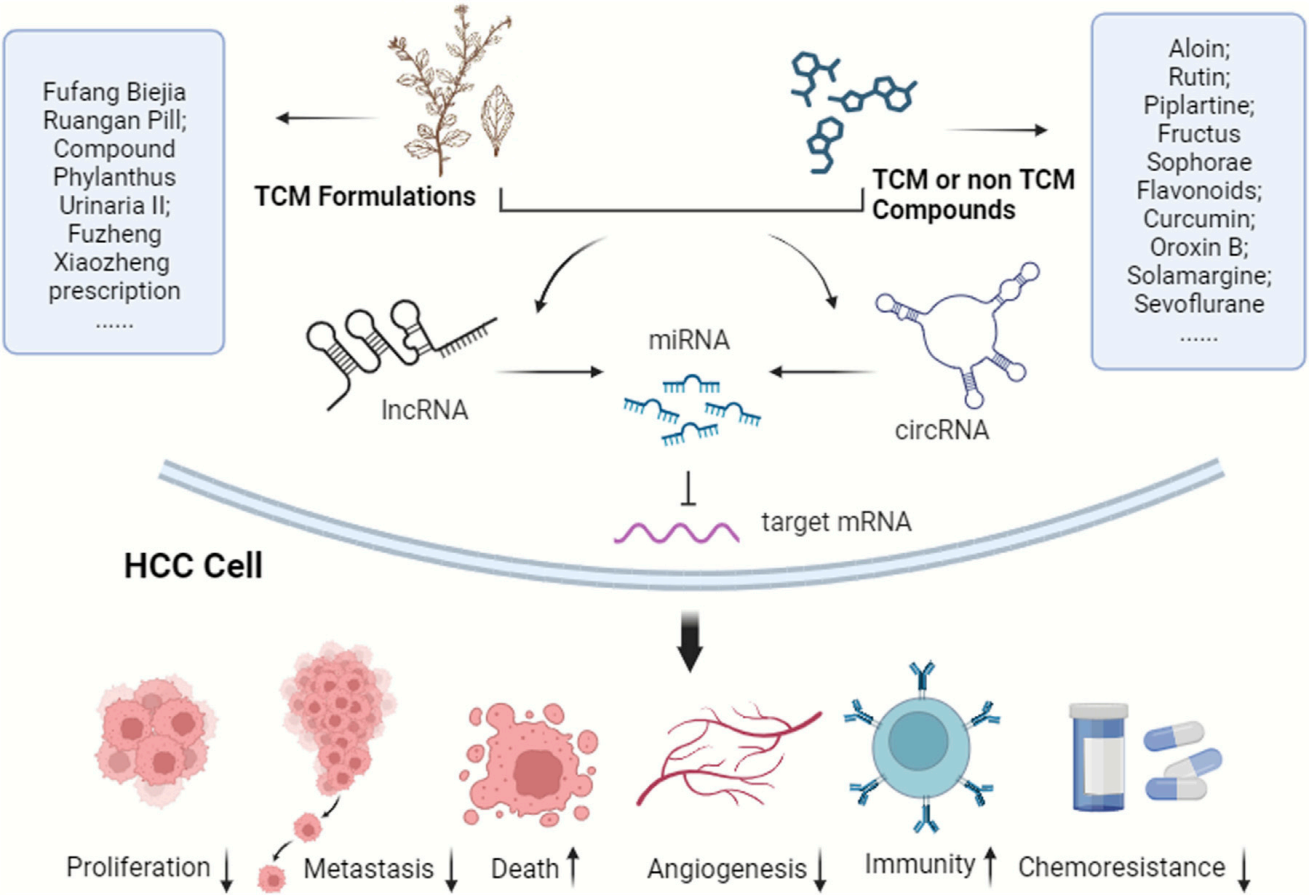
TCM and modern molecular compounds modulate the ceRNA network to inhibit HCC progression. These ingredients play crucial roles in various aspects, including suppressing HCC cell proliferation, invasion, and metastasis, inducing cell death, hindering angiogenesis, bolstering immunity, improving the tumor microenvironment, and reducing chemotherapy resistance.

Considering the pivotal role of the ceRNA network in HCC progression, it offers potential intervention targets for future HCC clinical treatments. As noted earlier, ncRNAs and their ceRNA networks are promising for drug-targeted therapy in HCC, attribute to their specific expression in HCC cells, reduced side effects, and regulatory targeting. TCM and modern molecular compounds have shown efficacy against HCC by influencing the ceRNA network (Figure 3). Currently, there is a scarcity of pharmacological research on treating HCC through ceRNA modulation. Research in TCM has mainly focused on active components rather than formulations, complemented by several studies on non-TCM products. With increasing insights into HCC’s epigenetic patterns, ceRNA networks are emerging as significant targets for novel drug development, particularly in TCM. Thus, the drugs have been categorized into two principal groups, TCM and non-TCM compounds, to elucidate their molecular mechanisms in HCC progression regulation via ceRNA networks.

### 6.1 TCM

The significant potential of TCM in HCC treatment necessitates an understanding of its mechanisms, particularly in complex herbal formulations. However, the complexity of these formulations and their multi-site, multi-target effects make it challenging to fully comprehend their therapeutic mechanisms through a single aspect or pathway. Advances in transcriptomics have revealed the involvement of ceRNA networks at various pathological stages of HCC, providing insights into HCC pathogenesis at the gene regulation level and presenting potential targets for TCM intervention. It has been established that ceRNA networks play a comprehensive role in TCM’s regulatory processes against HCC.

#### 6.1.1 TCM active compounds

Chinese compounds, key constituents of TCM, have garnered significant attention in pharmacological research for their varied pharmacological properties. TCM employs heat-clearing and detoxifying agents for effective cancer cell elimination, inflammation reduction, and tumor growth inhibition. *Scutellaria barbata* D. Don (SB) and *Oldenlandia diffusa* (Willd.) Roxb (OD), commonly used in TCM to treat tumors by clearing heat and toxins, have been shown to inhibit hepatitis B virus-associated HCC growth *in vitro* and *in vivo* by affecting circRNAs and related ceRNA networks. Their active components, particularly total flavonoids, lignans, and apigenin, played a pivotal role in ceRNA network regulation (Yang et al., 2021). Aloin, a compound found in aloe vera plant, could inhibit HCC cell malignant phenotype such as proliferation, invasion, and promote apoptosis and autophagy by regulating the circ0011385/miR-149–5p/WT1 axis (Fu et al., 2021). Other studies, including those on Solasonine, Curcumin, and Piplartine, demonstrated the potential of these TCM-derived active ingredients against HCC through the ceRNA network regulation, lncRNA CCAT1/miR-375–3p/SP1 (Liu et al., 2019b), circ0078710/miR-378b/PRIM2 (Chen et al., 2022b), and circ100338/miR-141–3p/ZEB1 (Cheng et al., 2020), respectively. Moreover, the ceRNA network involving lncRNA BANCR/miR-590–5p/OLR1 was implicated in the mechanism by which Rutin, a TCM active ingredient, mitigated sorafenib resistance in HCC (Zhou et al., 2021a). Furthermore, TCM active compounds frequently interact with parts of ceRNA networks. LncRNA GAS5 contained miR-21 response elements and competed with miR-21, upregulating its target genes PDCD4 and PTEN for anticancer effects (Hu et al., 2016). Studies indicated that resveratrol (Li et al., 2014), matrine (Luo et al., 2017), oxymatrine (Huang et al., 2014), and ursolic acid (Liu et al., 2015) all reduced miR-21 expression in HCC cells, inhibiting proliferation and promoting apoptosis, suggesting their involvement in the lncRNA GAS5/miR-21/PDCD4 pathway for anti-HCC action. Sophorae Fructus flavonoids regulated the lncRNA FBXL19-AS1/miR-342–3p axis, inhibiting HCC cell proliferation and invasion (Jin et al., 2020). LncRNA ATB bound competitively to the miR-200 family, elevating ZEB1 and ZEB2 levels, inducing EMT, and promoting HCC cell invasion (Yuan et al., 2014). HOTAIR contained MRE of miR-1 and miR-218 and negatively regulated the expression of miR-1 and miR-218 (Su et al., 2016), increased the levels of their target gene EZH2 (Fu et al., 2015), which played a carcinogenic role. Research has suggested that Curcumin and Curcumol’s anti-HCC mechanism may be closely related to the inhibition of miR-200 expression (Liang et al., 2013) and the HOTAIR/miR-218-2/EZH2 pathway (Tian et al., 2021), respectively. Oroxin B, a flavonoid from Oroxylum indicum (L.) Vent, demonstrated anti-HCC activity and improved HCC pathology by reducing miR-221 levels and deactivating the PTEN/PI3K/Akt pathway (Li et al., 2019). Meanwhile, the tumor-suppressive lncRNA CASC2 bound miR-221, influencing Caspase-3 expression and affecting HCC cell growth (Sun et al., 2022a). These studies illustrate the extensive role of ceRNA networks as targets in the anti-HCC mechanisms of TCM’s active ingredients.

#### 6.1.2 TCM formulations

Compared to individual herbs or active compounds, TCM formulations can exert a more synergistic and comprehensive effect on the body. In TCM practice, these formulations are essential. Numerous clinical and preclinical studies have validated the significant efficacy of TCM formulations in treating HCC. The Jiedu Quyu Kangai Recipe, for instance, has been shown to improve clinical parameters following hepatic artery embolization and to modulate the immune system (Zhang et al., 2020c). The Dahuang Zhechong Pill, when combined with transcatheter arterial chemoembolization (TACE), enhanced liver function, boosted immunity, reduced metastasis risk, and alleviated adverse reactions in HCC treatment (Dai et al., 2021). Similarly, the Qinggan Huadu Zhuyu Decoction significantly ameliorated clinical symptoms, bolstered immune function, and extended survival time in post-TACE patients (Shi et al., 2021a). Mechanistic studies on TCM formulations have explored various pathways, including inducing tumor cell apoptosis, autophagy, and ferroptosis, arresting the cell cycle, reducing proliferation, impairing telomerase activity, counteracting multidrug resistance in tumors, and remodeling the tumor microenvironment and immune responses (Lin et al., 2017; Wang et al., 2020). With the advent of ceRNA research, some studies have begun to examine the gene network regulatory mechanisms of TCM formulations. For example, the Fufang Biejia Ruangan Pills have been found to potentially disrupt the malignant progression from liver fibrosis to liver cancer by regulating the lncRNA TUG1/miR-328–3p/SRSF9 ceRNA regulatory axis (Liu, 2022). The Compound Phylanthus urinaria Ⅱ has been shown to inhibit HepG2 cell proliferation by modulating the ceRNA network lncRNA CCAT1/miR-let-7a/HMGA2/Cyclin D1 axis, thereby enhancing the inhibitory effect of miR-let-7a on HMGA2 and Cyclin D1 expression, manifesting its anti-HCC properties (Chen et al., 2021b). In our previous research, transcriptomic analysis and qRT-PCR validation revealed that the Fuzheng Xiaozheng prescription could inhibit HCC proliferation and reduce hepatic inflammation *via* circRNA and associated ceRNA networks (Liu et al., 2022a). Overall, while the modulation of the ceRNA network by TCM formulations shows promise, research in this area is still sparse and warrants further exploration in the future.

### 6.2 Non-TCM bioactive chemicals

In addition to TCM preparations, several natural compounds are known to regulate the ceRNA network. Three potential HCC therapeutic agents—decitabine, BW-B70C, and gefitinib—have been identified through big data mining and computational biology as promising for HCC treatment by modulating the circRNA-associated ceRNA network, though they require experimental validation (Xiong et al., 2018). Solamargine, a naturally occurring alkaloid, has been studied for its anti-HCC properties. It inhibited HCC growth and reversed sorafenib resistance *via* the ceRNA network HOTTIP-TUG1/miR-4726–5p/MUC1 (Tang et al., 2022b), and induced apoptosis and autophagy in HCC cells by suppressing the oncogenic factor LIF and modulating the miR-192–5p/CYR61/Akt signaling axis, repolarizing tumor-associated macrophages, and influencing other immune cells in the tumor microenvironment (Yin et al., 2022). Sevoflurane, a commonly used anesthetic, has been found to inhibit proliferation, invasion, and migration, and to promote apoptosis in HCC. Studies indicated that sevoflurane inhibited HCC progression by regulating the ceRNA networks circ0001649/miR-19a-3p/SGTB axis (Sun et al., 2023) and lncRNA KCNQ1OT1/miR-29a-3p CBX3 (Zhou et al., 2021b). Cyanidin-3-glucoside (C3G), extracted from *Morus alba* L., possesses notable antioxidant and antitumor activities. C3G could prevent diethylnitrosamine and acetamidofluorene-induced rat HCC precancerous lesions by regulating the autophagy protein ATG16L1 and its ceRNA network (circ0001345/miR-106b/ATG16L1), improving hepatic function and histopathological damage, thereby offering potential against HCC (Zabady et al., 2022). More detailed information on anti-HCC properties of TCM or non-TCM compounds is provided in Table 3.

**TABLE 3:** TCM compounds and non-TCM compounds experimentally demonstrated to inhibit HCC by modulating the ceRNA network.

Category	Drugs	CeRNA network	Binding sequences in ncRNA^a^	Functions on HCC	References
TCM (compounds)	Aloin	circ0011385/miR-149–5p/WT1	5’… GAGCCAGA … 3′	Inhibit HCC cell proliferation, invasion, tumor growth, and promote apoptosis and autophagy	Fu et al. (2021)
	Extracts of *Scutellaria barbata* D. Don (SB) and *Oldenlandia diffusa* (Willd.) Roxb	circ0008903, circ0000854, circ0001346, miR-107, miR-197–3p, miR-26a-5p*etc.*	N/A	Inhibit the growth of hepatitis-B-virus-associated HCC *in vitro* and *in vivo*	Yang et al. (2021)
	Solasonine	lncRNA CCAT1/miR-375–3p/SP1	5’…AACGAACA … 3′	Suppress the proliferation of HCC cells	Liu et al. (2019b)
	Rutin	lncRNA BANCR/miR-590–5p/OLR1	5’…AGCUCAGU … 3′	Mitigate sorafenib-induced autophagy and chemoresistance in HCC	Zhou et al. (2021a)
	Piplartine	circ100338/miR-141–3p/ZEB1	5’…CAGUGUU … 3′	inhibit cell proliferation of HCC	Cheng et al. (2020)
	Resveratrol, matrine, oxymatrine, ursolic acid	lncRNA GAS5/miR-21/PDCD4	5’…ACA​GGC​ATT​AGA​CAG​A-AAG​CTG … 3′	Inhibit HCC cell proliferation and promote apoptosis	Huang et al. (2014), Li et al. (2014), Liu et al. (2015), Luo et al. (2017)
	Flavonoids of Sophorae Fructus	lncRNA FBXL19-AS1/miR-342–3p	5’…GUU​GGG​AUU​AUA​GGU​GUG​AG … 3′	Inhibit the proliferation, migration and invasion of HCC cells	Jin et al. (2020)
	Curcumin	lncRNA ATB/miR-200/ZEB1/ZEB2; circ0078710/miR-378b/PRIM2	5’…CAGTGTT … 3’; 5’…CAAGUCCAG … 3′	Inhibit the invasion of HCC cells	Liang et al. (2013), Yuan et al. (2014), Chen et al. (2022b)
	Curcumol	lncRNA HOTAIR/miR-218-2/EZH2	N/A	Suppress HCC growth and metastasis	Tian et al. (2021)
	Oroxin B	lncRNA CASC2/miR-221/Caspase-3	5’…UACGUGGAAU … 3′	Induce apoptosis of HCC cells	Li et al. (2019), Sun et al. (2022a)
TCM (formulation)	Fufang Biejia Ruangan Pills	lncRNA TUG1/miR-328–3p/SRSF9	5’…AGGGCCA … 3′	Intervene in the "liver fibrosis to liver cancer" malignant transformation	Liu (2022)
	Compound Phylanthus urinaria Ⅱ	lncRNA CCAT1/miR-let-7a/HMGA2/Cyclin D1	N/A	Inhibit the proliferation of HepG2 cells	Chen et al. (2021b)
	Fuzheng Xiaozheng prescription	circRNA-4631, circRNA-4632, miR-136–5p, miR-181b-5p, miR-181d-5p, miR-376c-3p, miR-493–5p, miR-541–5p, miR-672–5p*etc.*	N/A	Inhibit HCC proliferation, attenuate hepatic inflammation, improve lipid and glucose metabolism	Liu et al. (2022a)
Non-TCM bioactive chemicals	decitabine, BW-B70C, gefitinib	circRNA104515, circRNA100291, miR-1303, miR-142–5p, miR-877–5p, miR-583, miR-1276, JUN, MYCN, AR, ESR1, FOXO1, IGF1, CD34	N/A	Potential therapeutic drugs to inhibit HCC	Xiong et al. (2018)
	Solamargine	lncRNA HOTTIP-TUG1/miR-4726–5p/MUC1	5’…UGC​ACG​UAA​GCC​UGG​CCC … 3′	Inhibit HCC growth and reversed sorafenib resistance, induce apoptosis and autophagy in HCC cells	Tang et al. (2022b)
	Sevoflurane	circ0001649/miR-19a-3p/SGTB; lncRNA KCNQ1OT1/miR-29a-3p/CBX3	5’…AGGTGGTGCT … 3′	Inhibit proliferation, invasion, migration, and promote apoptosis	Zhou et al. (2021b), Sun et al. (2023)
	Cyanidin-3-glucoside	circ0001345/miR-106b/ATG16L1	N/A	Improve the hepatic function and hepatic histopathological damage	Zabady et al. (2022)

^a^ Binding sequences of ncRNAs and miRNAs. N/A, not available, means that prediction and validation of the binding sequence was not done or was not provided in the study.

## 7 Discussion and conclusion

The progression from normal liver tissue to HCC is a lengthy and gradual process influenced by various factors, including hepatitis B or C virus infection, fat deposition, autoimmune disorders, alcoholism, and aflatoxin exposure. These factors can cause hepatocyte damage, followed by regeneration. However, persistent injury leads to continuous repair, eventually resulting in the development of fibrotic and proliferative nodules, precursors to HCC. As these hyperplastic nodules advance, characterized by liver lobular remodeling and abnormal cell proliferation, they may develop into overt HCC. Despite advancements in medical technology, the pathogenesis of HCC remains not fully understood. Therefore, investigating the regulatory mechanisms of HCC and identifying molecular targets for diagnosis, treatment, and prognosis are crucial for preventing and treating HCC.

As research into ncRNAs has deepened, genes like lncRNA and miRNA, once considered “noise”, have been increasingly recognized as pivotal in HCC development. The ceRNA network is extensively involved in various HCC pathological processes, including proliferation, migration, invasion, cell death, angiogenesis, and chemotherapy resistance. This review summarized the numerous ncRNAs and ceRNA networks involved in the development, diagnosis, and treatment of HCC, but most of them are only preliminary findings and the studies are superficial. Table 1, Table 2, Table 3 showed that certain ncRNAs played multiple roles, indicating that their roles have been widely confirmed and were better understood. LncRNAs such as TUG1 and NEAT1, along with their associated ceRNA networks, are extensively studied in HCC. They contributed significantly to HCC development, offering early diagnostic value and serving as targets in drug-regulatory mechanisms, potentially representing some of the most important ncRNAs in HCC (Bu et al., 2020; Farzaneh et al., 2022). TUG1, first identified in the retina of taurine-treated mice, is engaged in a variety of physiopathological processes involving the cardiovascular, respiratory, digestive, and nervous systems, and has important regulatory roles in a variety of tumor cells (Guo et al., 2020). TUG1 is located on chromosome 22q12.2 and includes 4 exons. Although TUG1 has 20 transcripts, 19 of them do not have the ability to encode proteins (Farzaneh et al., 2022). Previous studies have shown that TUG1 played an oncogenic role in tumors such as colorectal (Ibrahim et al., 2023), prostate (Yu et al., 2019), bladder (Tan et al., 2022), lung (Li et al., 2021a), oral squamous cell (Jiang et al., 2022), and ovarian cancers (Zhan et al., 2020), as well as being involved in the pathogenesis of non-cancerous diseases such as renal ischemia-reperfusion injury (Sun et al., 2022b), pulmonary fibrosis (Qi et al., 2023), and myocardial infarction (Dang et al., 2023). Thus, TUG1 could act as a pathogenic factor in a tissue-specific or environment-specific manner. Moreover, TUG1 performed a critical role in HCC by targeting multiple ceRNA networks. For example, TUG1/hsa-miR-582–5p/Siglec-15 contributed immunosuppression in HCC (Ren et al., 2022), TUG1/miR-137/AKT2 promoted the EMT process (Li et al., 2021d), TUG1/miR-335–5p/CXCR4 facilitated the activation of Wnt signaling pathway (Xie et al., 2020), TUG1/miR-328–3p/SRSF9 caused malignant progression of HCC (Liu Y. et al., 2022d) and was effectively inhibited as a target of prescription FufangBiejia Ruangan Pill. In brief, TUG1 could be a potential target for the diagnosis and prevention of HCC. As well, NEAT1, a lncRNA enriched in the nucleus, was discovered in 2007 in the paracrine cristae (Hutchinson et al., 2007). It has two transcripts, an unspliced non-coding transcript approximately 4 kb in length, and a lower-expression isoform greater than 17 kb. NEAT1 is localized to the pars compacta and has been extensively studied in a variety of cancers and its role as an oncogenic factor has been confirmed (Klec et al., 2019). Its mediated ceRNA network could promote the growth of HCC cells by inhibiting their senescence (Chen et al., 2023), regulating aerobic glycolysis (Zhang et al., 2022b), modulating sorafenib resistance (Niu et al., 2020), enhancing radioresistance in HCC (Chen and Zhang, 2019), and inhibiting the function of CD8^+^T cells (Yan et al., 2019). However, the most promising aspect of the current study is its early diagnosis and prognosis determination of HCC, and it has been elucidated in this review that the combined diagnostic model of NEAT1 in combination with TUG1 and AFP significantly outperformed the diagnostic efficiency of using AFP alone (Mohyeldeen et al., 2020). NEAT1 was significantly elevated in the serum of HCC patients, and ROC curve analysis showed AUC = 0.981, *p* < 0.001, sensitivity 100%, specificity 88.9% (Shaker et al., 2019). Additionally, The lncRNA CCAT1 is a typical pro-carcinogenic gene in HCC (Zhang et al., 2019), and is also repeatedly mentioned throughout this review. CCAT1 was first identified in colon cancer and mapped to chromosome 8q24.2. The CCAT1 gene encodes two isozymes, the short isoform CCAT1-S and the long isoform CCAT1-L. CCAT1-L is highly expressed mainly in the nucleus, whereas CCAT1-S is mainly found in the cytoplasm, and a positive correlation is likely to exist between the two (Liu et al., 2019a). The CCAT1-associated ceRNA network was involved in HCC cells autophagy (Guo et al., 2019), chemotherapeutic resistance (Xia et al., 2022), and promotion of tumor-associated macrophage polarization (Deng et al., 2020), and bound to C-Myc as an independent risk factor for HCC patients (Zhu et al., 2015). Furthermore, its related ceRNA network could be modulated by TCM and chemical drugs to produce anti-HCC effects, underscoring its research significance in HCC in this review. Alternatively, several target genes within the ceRNA network are noteworthy. The FOX gene family, regulated by various ncRNAs, and its related ceRNA networks such as lncRNA MCM3AP-AS1/miR-194–5p/FOXA1, lncRNA MFI2-AS1/miR-134/FOXM1, lncRNA MALAT1/miR-3064–5p/FOXA1, and circFBXO11/miR-605/FOXO3, played significant roles in HCC’s biological processes like proliferation, invasion, metastasis, and drug resistance, suggesting that FOX genes may be key targets for HCC prevention and treatment.

The discovery of key ncRNAs and ceRNA networks in HCC has broadened the scope of research beyond specific pathways, offering a comprehensive view of HCC’s malignant progression from a gene regulation standpoint. This paradigm shift has enhanced our understanding of HCC’s complex pathology, introducing new perspectives into its pathogenesis and enriching the research field. However, the study of ncRNAs is still nascent compared to that of protein-coding genes. While numerous specific ncRNAs and ceRNA networks have been identified in HCC, their functions are not fully understood, and their role remains confined to molecular expression levels. This limitation hinders the identification of ncRNAs with critical roles in HCC development. Furthermore, due to incomplete understanding, methods for detecting and characterizing functional ncRNAs are underdeveloped and need further refinement.

Research on the molecular mechanisms governing ceRNA network regulation in HCC is just beginning. Although the functions of ncRNAs and their role as ceRNA sponges are increasingly recognized, most studies are conducted at the cellular level using overexpression and knockdown assays. The variations in ceRNA interactions across different stages, populations, and their contributions to tumor aggressiveness and therapeutic resistance are yet to be clarified. Additionally, the human body’s complexity and various factors that can affect ceRNA activity, such as cellular localization, concentration of ceRNA components, interactions with RNA-binding proteins, RNA transcription, and ceRNA affinity in endogenous cellular environments, necessitate more preclinical experiments and clinical trials (Shi et al., 2021b). Moreover, while current research often depicts ceRNA interactions as singular, the existence of large, interconnected networks suggests significant secondary and indirect interactions that could substantially impact ceRNA regulation, warranting further investigation. Therefore, integrating advanced single-cell technologies, bioinformatics, and computer-aided modeling may elucidate the role of ceRNA interactions in tumor heterogeneity, offering profound clinical insights.

CeRNA networks, due to their impact on various aspects of HCC progression, present novel biomarkers or targets for HCC diagnosis and treatment. They have also elucidated the molecular mechanisms underlying the anticancer effects of drugs, particularly TCM, which is known for its multi-target, multi-process, and multi-effect approach. This insight offers a new perspective on the mechanistic study of TCM in HCC treatment, enhancing basic research and its application prospects. However, studies exploring drug modulation mechanisms within the ceRNA network are limited, possibly due to the nascent state of ceRNA research. Current investigations primarily focus on active compounds or specific segments of the ceRNA network. Concentrating solely on a single Chinese herb or an active component is inconsistent with the holistic principles of TCM, and examining a singular part of the ceRNA network overlooks the broader gene transcription interactions. Systematic research into TCM formulations through ceRNA networks remains underdeveloped. This gap can be attributed to the complexity of multi-ingredient herbal formulations, difficulty in identifying specific drug targets, and challenges inherent in TCM formulations, all of which pose significant challenges in TCM research. The diverse chemical components of TCM generate multiple biological effects through their interactions, complicating the identification of their material basis and action targets against HCC. Future research should, therefore, leverage modern techniques guided by TCM theory to unravel the ceRNA action mechanisms of TCM formulations against tumors. Employing genomics, transcriptomics, and bioinformatics will enable a comprehensive analysis of the ceRNA network mechanisms of TCM components in HCC intervention, systematically elucidating TCM’s material basis against HCC. As modern technology continues to advance, the roles of ceRNA regulation in HCC development and the mechanisms by which TCM treats HCC will become increasingly clear.

In conclusion, advancements in gene epigenetic technology have expanded research into ncRNAs, uncovering the regulatory mechanisms of the ceRNA network. Present research trends indicate that lncRNA TUG1, lncRNA NEAT1, and lncRNA CCAT1 are among the most extensively studied ncRNAs in HCC, owing to the significant role of their associated ceRNA networks, as confirmed by numerous studies. These insights not only enhance our understanding of HCC’s pathogenesis and progression from a gene transcriptional regulation standpoint but also pave the way for novel diagnostic, monitoring, and treatment approaches for HCC. Most notably, ceRNAs offer therapeutic targets for cancer treatment. Investigating the anticancer mechanisms of TCM or molecular compounds through the lens of the ceRNA network aids in systematically elucidating the material basis of TCM and its active components. However, it is imperative to integrate more contemporary medical technologies and undertake animal or clinical trials to explore the *in vivo* interactions of the ceRNA network and the systematic regulation of the ceRNA network by drugs, including TCM.

## Abbreviations

AFP, Alpha-fetoprotein; AFP-L3, fetoprotein lectin 3; ATG3, autophagy-related gene 3; ATG5, autophagy-related gene 5; C. elegans, Caenorhabditis elegans; ceRNA, competing endogenous RNA; CT, computer tomography; circRNA, circular RNA; C3G, cyanidin-3-glucoside; DCP, des-gamma-carboxyprothrombin; EMT, epithelial-mesenchymal transition; GPC3, glypican-3; HCC, hepatocellular carcinoma; lncRNA, long non-coding RNA; miRNA, microRNA; MRI, magnetic resonance imaging; MRE, miRNA response elements; ncRNA, non-coding RNA; NK cells, natural killer cells; NF-κB, nuclear factor-kappa B; OD, *Oldenlandia diffusa* (Willd.) Roxb; OLR1, oxidized low-density lipoprotein receptor 1; SB, *Scutellaria barbata* D. Don; TCM, traditional Chinese medicine; TME, tumor microenvironment; TACE, transcatheter arterial chemoembolization; VEGF, vascular endothelial growth factor; 3'UTR, 3' untranslated region.
