# Supplementary figures and images for: The role of competing endogenous RNA network in the development of hepatocellular carcinoma: potential therapeutic targets
Source: Front Cell Dev Biol. 2024 Jan 31;12:1341999. doi: 10.3389/fcell.2024.1341999 (PMC10864455; doi:10.3389/fcell.2024.1341999)

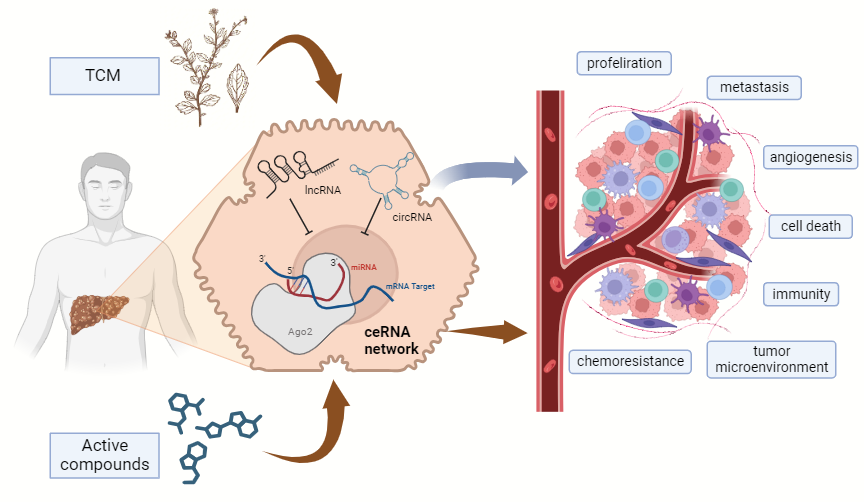

Supplement: Supplementary file 1 [file Image1.TIF]
